# Severe *Pneumocystis jirovecii* pneumonia in an idiopathic CD4^+^ lymphocytopenia patient: case report and review of the literature

**DOI:** 10.1099/jmmcr.0.T00017

**Published:** 2014-12-01

**Authors:** Bernabé F. F Chumpitazi, Pierre Flori, Jean-Baptiste Kern, Marie-Pierre Brenier-Pinchart, Sylvie Larrat, Clémence Minet, Laurence Bouillet, Danièle Maubon, Hervé Pelloux

**Affiliations:** ^1^​ Laboratory of Parasitology – Mycology, Grenoble University Hospital, CS 10217, F-38043 Grenoble, France; ^2^​ Laboratory of Parasitology – Mycology, University Hospital of Saint Etienne, Saint Etienne, Av Albert Raimond, F-42055 Saint Etienne, France; ^3^​ Laboratory of Virology, Grenoble University Hospital, CS 10217, F-38043 Grenoble, France; ^4^​ Intensive Care Unit, Grenoble University Hospital, CS 10217, F-38043 Grenoble, France; ^5^​ Internal Medicine, Grenoble University Hospital, CS 10217, F-38043 Grenoble, France; ^6^​ Université de Grenoble Alpes, Grenoble, France; ^7^​ Jean Monnet University, Saint Etienne, France

**Keywords:** Idiopathic CD4^+^ lymphocytopenia, opportunistic infections, trimethoprim-sulfamethoxazole

## Abstract

**Introduction:**

When diagnosing *Pneumocystis jirovecii* pneumonia (PJP), the clinical suspicion must be confirmed by laboratory tests. PJP is rarely described in patients with idiopathic CD4^+^ lymphocytopenia (ICL), a rare T-cell deficiency of unknown origin with persistently low levels of CD4^+^ T-cells (<300 µl^−1^ or <20 % of total lymphocytes) but repeated negative human immunodeficiency virus (HIV) tests. We retrospectively analysed a case of an ICL patient with severe PJP associated with multiple opportunistic infections (OIs). We also reviewed the literature since 1986.

**Case presentation:**

A laboratory-confirmed case of PJP associated with invasive candidiasis and cytomegalovirus infection was reported in an ICL patient. Despite early treatment, the patient died of respiratory failure under polymicrobial pneumonia. According to the literature, the mortality rate of ICL patients is 10.4 % (33/316). In ICL patients, the risk of OI is 83.2 % (263/316), with viral infections being the most prevalent (58.2 %, 184/316), followed by fungal infections (52.2 %, 165/316) and mycobacterial infections (15.5 %, 49/316). Dysimmunity is reported in 15.5 % (49/316) of ICL patients. Among the fungal infections, cryptococcal infections are the most prevalent (24.1 %, 76/316), followed by candidiasis (15.5 %, 49/316) and PJP (7.9 %, 25/316).

**Conclusions:**

The high risk of OIs underlines the importance of more vigorous preventative actions in hospitals. The response to therapy and the detection of early relapse of PJP may be monitored by several laboratory tests including quantitative PCR. It is essential to treat the ICL and to follow the guidelines concerning therapy and prophylaxis of OIs as given to HIV patients.

## Introduction

*Pneumocystis jirovecii* pneumonia (PJP) is infrequently reported in idiopathic CD4^+^ lymphocytopenia (ICL) patients (Ahmad *et al*., 2013). ICL is a rare and heterogeneous T-cell immunodeficiency syndrome of unknown origin with repeatedly low levels of CD4^+^ T-cells (<300 µl^−1^ or <20 % of total lymphocytes), and no evidence of human immunodeficiency virus (HIV) infection (Régent *et al.*, 2014; Smith *et al.*, 1993).The differential diagnosis of ICL remains a challenge as it involves a wide range of analyses in different specialty areas such as immunology, haematology, rheumatology and infectious diseases (Zonios *et al*., 2012). Moreover, the differential diagnosis is also complicated by the fact that ICL patients may have underlying AIDS (Smith *et al*., 1993). At the time when multiple opportunistic pathogens are identified, the CD4^+^ counts are generally ≤150 cells µl^−1^ (Ahmad *et al.*, 2013; Denis *et al.*, 2014; Marukutira *et al.*, 2014). We report a case of PJP associated with cytomegalovirus (CMV), *Candida parapsilosis*, *Streptococcus pneumoniae* and *Pseudomonas aeruginosa* infection. The aim of the present review was to place our rare PJP case in perspective and to evaluate the prevalence of opportunistic infections (OIs) in ICL patients, in particular the importance of fungal infections.

## Methods

This was a retrospective case study. Bronchoalveolar lavage fluid (BALF) samples from the ICL patient were used for *in vitro* culture for *Candida*, microscopy for *Pneumocystis* identification and quantitative PCR (q-PCR) for the detection of *P. jirovecii* DNA; candidaemia was also evaluated in this patient (Chumpitazi *et al.*, 2011, 2014; Fricker–Hidalgo *et al.*, 2004). The q-PCR target used was the gene encoding the *Pneumocystis* major surface glycoprotein (MSG) (Chumpitazi *et al.*, 2011; Cushion & Stringer, 2010; Khot & Fredricks, 2009). The amplicon DNA was sequenced, and pair-wise sequence alignments of the amplicons were performed using ceq2000 DNA analysis software (Beckman). Further identification of MSG genes of *P. jirovecii* was performed by blast from both nucleotide and amino acid sequences of the three successive isolates from BALF samples with at least 99 % nucleotide identity for each.

For the review of the literature, a Medline search was performed using the keyword combinations: ‘idiopathic CD4^+^ lymphocytopenia’ and ‘opportunistic infections’, or ‘idiopathic CD4^+^ lymphocytopenia’ and ‘*Pneumocystis* pneumonia’. We found a systematic review of ICL from 1986 to April 2012 (Ahmad *et al*., 2013). We added to this review the studies from May 2012 to April 2014.

## Case report

A 52-year-old man, with a medical history of surgery to remove an eyelid basocellular carcinoma, presented with dyspnoea on exertion and dry cough without fever at day −3. The patient had hypogammaglobulinaemia, repeated negative HIV tests and autoimmune serologies and was referred to the intensive care unit with asthenia, dyspnoea, dry cough and low-grade fever (day 0). Care of the patient required mechanical ventilation for 38 days. The patient had lymphopenia and low CD4^+^ counts (≤70 cells µl^−1^) during the follow-up (Table 1). The diagnosis of PJP, which revealed the patient’s ICL (Smith *et al*., 1993), was confirmed by microscopic analysis of a BALF sample at day 4 and the patient was treated with trimethoprim-sulfamethoxazole (TMP-SMZ). At day 15, the *Pneumocystis* infection resolved, but the patient presented with severe pancytopenia. TMP-SMZ treatment was stopped and pentamidine isethionate was prescribed at day 16. However, as relapse of PJP was confirmed by q-PCR (day 19) and by microscopy (day 24), TMP-SMZ was reintroduced from day 24 to day 51 (Table 1). To assess the response to therapy, microscopic analysis and q-PCR were performed on BALF samples (Table 1) (Chumpitazi *et al*., 2011). In a context of multiple polymicrobial pneumonia from day 4 to day 46 (e.g. invasive candidiasis, CMV viraemia and *P. aeruginosa* infection from day 28 to day 34), the specificity of the *Pneumocystis* q-PCR used was confirmed by the amplicon nucleotide sequence and blast analysis. The q-PCR from BALF was positive for three analysed samples (Table 1). The MSG genes/amino acid sequences identified were AF033209.1/AAC34972.1, AF033210.1/AAC34973.1, AF372980.1/AAL23912.1, DQ000981.1/AAY18808.1 and DQ000983.1/AAY18810.1 (GenBank accession numbers). The first three MSG genes were common to all isolates. At day 51, the patient died of respiratory failure in the context of severe immunodeficiency.

## Review

Briefly, Table 2 summarizes the review of the literature on ICL and OIs from 1986 to April 2014. The last column of Table 2 indicates the number of PJP cases described, including the ICL case reported here. Among the ICL patients, the risk of OIs was 83.2 % [263/316; 95 % confidence interval (CI) 78.7–87.1 %). Viral infections had a prevalence of 58.2 % (184/316; 95 % CI 52.7–63.6 %), followed by fungal infections at 52.2 % (165/316; 95 % CI 46.7–57.7 %), and mycobacterial infections at 15.5 % (49/316; 95 % CI 11.8–19.9 %). The prevalence of protozoan infections was 3.5 % (11/316; 95 % CI 1.8–6.1 %). Among the fungal infections, cryptococcal infections were the most prevalent at 24.1 % (76/316), followed by candidiasis at 15.5 % (49/316) and PJP at 7.9 % (25/316). Among the viral infections, human papillomavirus (HPV) was the most prevalent at 12.7% (40/316; 95 % CI 9.3–16.8 %), followed by varicella-zoster virus (VZV) at 10.8 % (34/316; 95 % CI 7.7–14.7 %), herpes simplex virus types 1 and 2 (HSV-1 and HSV-2) at 7.0 % (22/316; 95 % CI 4.5–10.39 %) and CMV at 5.1 % (16/316; 95 % CI 3.0–8.1 %). Dysimmunity was reported in 15.5 % (49/316; 95 % CI 11.8–19.9 %) of ICL patients. The more prevalent autoimmune diseases described were Sjögren’s syndrome, sarcoidosis and psoriasis (Ahmad *et al.*, 2013; Baroudjian *et al.*, 2014). Malignancies were reported in 17.7 % (53/300; 95 % CI 13.6–22.4 %) of ICL patients (Ahmad *et al.*, 2013; Ollé–Goig *et al.*, 2012; Régent *et al.*, 2014). The more prevalent malignancies cited were squamous and basal cell carcinoma of the skin, lymphomas, Kaposi’s sarcoma and Bowen’s disease.

Taking all the reports in the literature, the mortality rate of ICL patients was 10.4 % (33/316; 95 % CI 7.4–14.3 %) (Thoden *et al.*, 2013; Zonios *et al.*, 2012). The mortality rate may depend on the type of OI; for example, the mortality rate was 46.6 % (7/15) in patients with HSV encephalitis and 72.7 % (8/11) in HIV patients with bacterial meningitis in whom CMV was detected (Ghannad *et al.*, 2013; Kelly *et al.*, 2012). The mortality rates related to invasive candidiasis varied from 33.7 to 75.0 %, to cryptococcal infection from 17 to 27 %, and to PJP from 10 to 12 % (Azie *et al.*, 2012; Chumpitazi *et al.*, 2014; Miller *et al.*, 2013; Pappas, 2013).

## Discussion

The differential diagnosis of ICL is difficult given its scarcity and the extensive number of analyses that need to be performed in different specialties (Zonios *et al*., 2012). By the time an OI is identified, the CD4^+^ counts are generally ≤150 cells µl^−1^ and the patient has become susceptible to multiple opportunistic pathogens (Ahmad *et al.*, 2013; Denis *et al.*, 2014; Marukutira *et al.*, 2014). As seen in the present review, the risk of OIs in ICL patients is very high, and prophylaxis against these pathogens is essential. This high risk of OIs emphasizes the requirement for more vigorous preventative measures to be taken in hospitals (Walzer, 2013). Cryptococcosis has been described as the most common opportunistic disease in ICL patients; however, mycobacterial disease and progressive multifocal leukoencephalopathy are also mentioned (Ahmad *et al.*, 2013; Zonios *et al.*, 2007, 2012). Other OIs may also occur such as tuberculosis, histoplasmosis and dermatomal VZV (Duncan *et al.*, 1993; Luo & Li, 2008; Zonios *et al.*, 2007, 2012). The clinical presentation of PJP can vary from one patient to another, although common characteristics are present such as progressive dyspnoea, dry cough, hypoxia and fever. Chest radiography and high-resolution assisted tomography can give valuable data in the diagnosis of *Pneumocystis* pneumonia. However, the clinical suspicion of OI needs to be confirmed by further laboratory analyses, such as *in vitro* culture, microscopic observation and q-PCR from BALF samples, as other invasive fungal infections may occur, as in the present case. Amplicon nucleotide sequences after PCR and blast analysis may provide a new method to validate the suspicion of clinical PJP.

The particularity of our case report resided in severe PJP complicated by viral, bacterial and fungal infection, which is a lethal and very rare event in patients with ICL. A marker of PJP severity and poor prognosis was the high serum concentrations of C-reactive protein (CRP), which were in the range of those found elsewhere in PJP patients (Sage *et al.*, 2010). Venzor *et al*. (1997) described the case of an ICL patient with PJP, CMV and *Candida* infection. During the follow-up, the common factor of polymicrobial pneumonia between this case and ours was the CD4^+^ counts of ≤70 cells µl^−1^. This suggests that multiple OIs may occur below this CD4^+^ count cut-off. The misdiagnosis of PJP and invasive candidiasis may have fatal consequences in ICL patients given its low prevalence and the diagnostic dilemmas it poses.

In our clinical case, the *Pneumocystis* MSG load (at day 42) was in favour of renewed infection and failure of the treatment with TMP-SMZ. The limit of the q-PCR used at day 42 was mainly the differentiation of active *P. jirovecii* infection and residual *Pneumocystis* colonization, which we confirmed here by amplicon nucleotide sequence and blast analysis in the absence of a positive microscopic observation (Alanio *et al.*, 2011; Chumpitazi *et al.*, 2011; Roux *et al.*, 2014). We suggest that both q-PCR and microscopy are used to assess the response to therapy and to detect an eventual relapse early on. PJP relapse is seldom described in the literature. Only one out of 91 PJP patients had a severe relapse (1.1 %; 95 % CI 0.3–5.9 %) (Duncan *et al.*, 1993; Kaczmarski *et al.*, 1994; Matsuyama *et al.*, 1998; Sinicco *et al.*, 1996; Venzor *et al.*, 1997; Zicklerova *et al.*, 2012; Zonios *et al.*, 2007). Adverse effects of TMP-SMZ were reported in 198 out of 1188 cases (16.7 %) (Helweg–Larsen *et al*., 2009). Pentamidine prophylaxis was prescribed after the severe pancytopenia due to TMP-SMZ, as microscopic observation of the BALF sample became negative for *P. jirovecii* at that time. Alternative therapies include a low dose of TMP-SMZ associated with caspofungin, dapsone plus trimethoprim, atovaquone or clindamycin administered with primaquine and pentamidine (Castro & Morrison–Bryant, 2010; Esteves *et al.*, 2014; Helweg–Larsen *et al.*, 2009; Tu *et al.*, 2013). However, the development of new molecules against OIs having high efficacy and minimum side effects for CD4 cell recovery is required.

To decrease the number of potential OIs, it is essential to treat idiopathic CD4^+^ lymphocytopenia as has been done for HIV patients, using an appropriated antiviral therapy. With this aim, several treatments have been proposed for ICL patients such as IL-2, IL-7 and IFN-γ (Régent *et al.*, 2012, 2014; Zonios *et al.*, 2012). Bone-marrow transplantation may also be an optional treatment for normal recovery of CD4^+^ counts (Zonios *et al*., 2012). ICL patients may also have AIDS-related diseases, and in this case the guidelines and/or expert opinions concerning therapy and prophylaxis of opportunistic infections given for HIV patients should also be applied to ICL patients (Masur *et al.*, 2014; Thoden *et al.*, 2013).

**Table 1.** Treatment and biological monitoring of an ICL patient with PJP complicated by viral, bacterial and fungal infections. The ICL patient had lymphopenia, high serum concentrations of C-reactive protein (CRP) and lactate dehydrogenase (LDH), plus low CD4^+^ counts. CRP levels diminished after treatment but remained relatively high.

Time	Lymphocytes (Giga l^−1^)#	CD4^+^ (cells µl^−1^)	CRP (mg l^−1^)	LDH (IU l^−1^)	Microscopy*	MSG (copies ml^−1^)†	Other pneumonia agent
Days 0–6	0.4	10	193/229	1124	+++	nd	CMV
Days 7–13	0.0/0.3	70	59/130	1558	−	nd	CMV
Days 14–20	0.0/0.3	nd	43/81	nd	−/−	3 ×10^2^ (PCC-1)	CMV; *S. pneumoniae*
Days 21–27	0.0/0.1	10	160	609	++	2.5 ×10^6^ (PCC-2)	CMV
Days 28–34	0.1	nd	201	952	−	nd	CMV; *C. parapsilosis; P. aeruginosa*
Days 35–41	0.5	nd	38/105	nd	−	nd	*P. aeruginosa*
Days 42–46	0.3	nd	136	nd	−/−	4.7 ×10^8^ (PCC-3)	*P. aeruginosa*

^#^When two values are given, it represents two different measures in the cited period.

< ast?> Microscopic analysis of BALF samples: +++, first PJP episode: numerous cysts and trophozoites of P. jirovecii observed at day 4; ++, the PJP relapsed under pentamidine: numerous cysts and trophozoites of P. jirovecii observed at day 24; −, single negative observation in the cited period; −/−, two negative observations in the cited period.

**< dagger?>:** The q-PCR detected relapse of PJP (day 19) earlier than the microscopy (day 24). The patient died at day 51.

ND, Not done. PCC-1, PCC-2 and PCC-3: MSG amplicon nucleotide sequences at D-15, D-19 and D-42, respectively.

**Table 2.** Review of the literature of ICL patients with opportunistic infections

Study (no. patients)	Age [years (range)]	CD4^+^ [cells µl^−^^1^ (range)]	Disease or agent (no. cases)	PJP (no. cases)
Ahmad *et al*., 2013 (review 1989–April 2012) (*n* = 259)	41 (1–85)	143 (10–293)	Cryptococcosis (69); *Mycobacterium* (44); candidiasis (42); autoimmune (37); VZV (34); HPV (30); HSV-1 and -2 (21); CMV (15)	20
Cohen *et al*., 2012 (*n* = 1)	48	Very low	Cryptococcosis and liver injury	1
Colomba *et al*., 2012 (*n* = 1)	38	138 (138–169)	*Mycobacterium* (1)	0
Chen *et al*., 2012 (*n* = 6)	47 (17–80)	96 (12–179)	Cryptococcosis (6)	0
Gonzalez-Estrada & Fernandez, 2013 (*n* = 1)	40	8	*Mycobacterium*	1
Ollé-Goig *et al*., 2012 (*n* = 2)	nd	<300	Tuberculosis (1); Kaposi’s sarcoma (1)	0
Régent *et al*., 2014 (*n* = 40)	44 (19–70)	127 (4–294)	Autoimmune (14); HPV (12); cryptococcosis (4); *Candida* (6)	2
Baroudjian *et al*., 2014 (*n* = 4)	nd	<300	Autoimmune (4)	0
McBath *et al*., 2014 (*n* = 1)	26	<300	West Nile virus (1)	0
This study (*n* = 1)	52	10 (10–70)	CMV; *Candida*	1
Total (*n* = 316)	41 (1–85)	140 (4–294)	OIs (263)	25

## Abbreviations

CI: confidence interval
BALF: bronchoalveolar lavage fluid
CMV: cytomegalovirus
CRP: C-reactive protein
HIV: human immunodeficiency virus
HPV: human papillomavirus
HSV: herpes simplex virus
ICL: idiopathic CD4^+^ lymphocytopenia
MSG: *Pneumocystis* major surface glycoprotein
OI: opportunistic infection
PCC-1, PCC-2 and PCC-3: MSG amplicon nucleotide sequences from BALF samples at D-15, D-19 and D-42: respectively
PJP: *Pneumocystis jirovecii* pneumonia
q-PCR: quantitative PCR
TMP-SMZ: trimethoprim-sulfamethoxazole
VZV: varicella-zoster virus
